# Supplementary material for: DGCR8 deficiency impairs macrophage growth and unleashes the interferon response to mycobacteria
Source: Life Sci Alliance. 2021 Mar 26;4(6):e202000810. doi: 10.26508/lsa.202000810 (PMC8008949; doi:10.26508/lsa.202000810)
Supplement: Supplementary file 6 [file LSA-2020-00810_Table_S6.docx]

Supplementary Table S6

Primers and probes used for qRT-PCR

| **gene symbol** | **primer number** | **probe** | **primer sequence** | **alternative name** |
| --- | --- | --- | --- | --- |
| CCL2 | RL473 | 22 | tcactgaagccagctctctct |  |
|  | RL474 |  | gtggggcgttaactgcat |  |
| CCL3 | RL412 | 22 | tctgtcacctgctcaacatca |  |
|  | RL413 |  | cggggtgtcagctccata |  |
| CCL4 | RL557 | 1 | gccctctctctcctcttgct |  |
|  | RL558 |  | ggagggtcagagcccatt |  |
| CD69 | RL730 | 29 | ggaaaatagctcttcacatctgg |  |
|  | RL731 |  | tgatgcttctcaaaatgtatactgg |  |
| CSF3 | RL559 | 67 | cctggagcaagtgaggaaga |  |
|  | RL560 |  | cagcttgtaggtggcacaca | G-CSF |
| CXCL10 | RL 724 | 3 | gctgccgtcattttctgc |  |
|  | RL725 |  | tctcactggcccgtcatc |  |
| DGCR8 | RL1452 | 99 | ggatgaagaggccttgaattt |  |
|  | RL1453 |  | tcctccattcgcctcttct |  |
| HPRT | RL415 | 95 | tcctcctcagaccgctttt |  |
|  | RL416 |  | cctggttcatcatcgctaatc | HPGRT1 |
| IFIT2 | RL782 | 68 | gcaagatgcaccaagatgag |  |
|  | RL783 |  | cttctaatgaagtgctccagacc |  |
| IFNβ | RL469 | 22 | tattgttgtacgtctcctggatg |  |
|  | RL470 |  | ttgcttttcctctagtactgtcttca |  |
| iNOS | RL453 | 13 | ctttgccacggacgagac |  |
|  | RL454 |  | tcattgtactctgagggctgac | Nos2 |
| IL-6 | RL409 | 6 | gctaccaaactggatataatcagga |  |
|  | RL410 |  | ccaggtagctatggtactccagaa |  |
| ISG15 | RL1700 | 102 | gaacaagtccacgaagaccag |  |
|  | RL1701 |  | gcagctccttgtcctccat |  |
| MINCLE | RL1216 | 15 | gctcacctggtggttatcg |  |
|  | RL1217 |  | aggttttgtgcgaaaaagga | Clec4e |
| MMP9 | RL1638 | 19 | agacgacatagacggcatcc |  |
|  | RL1639 |  | tcggctgtggttcagttgt |  |
| RIG-I | RL1688 | 67 | gaacaaaccgggcaacag |  |
|  | RL1689 |  | ttgttatctccgctggctct | Ddx58 |
| Serpinb2 | RL1751 | 76 | atcaccacaagaaacccagag |  |
|  | RL1752 |  | ggattttatctcctgcttgtgc |  |
| TNF | RL423 | 25 | ctgtagcccacgtcgtagc |  |
|  | RL424 |  | ttgagatccatgccgttg | TNF-α |
